# Clinico-epidemiological profile and risk factors of hypertensive crisis among patients attended at a tertiary care hospital in Somalia

**DOI:** 10.1038/s41598-023-27683-4

**Published:** 2023-01-09

**Authors:** Mohamed Farah Yusuf Mohamud

**Affiliations:** Mogadishu Somali Turkish Education and Research Hospital, Thirty Street, Alikamin, Wartanabada District, Mogadishu, Somalia

**Keywords:** Cardiology, Cardiovascular biology

## Abstract

Hypertensive crisis (HC) is a life-threatening clinical condition in which an abrupt rise in arterial blood pressure can lead to acute damage to vital organs. The main objective of our study is to determine the epidemiological profile, clinical characteristics, and risk factors of hypertensive crisis patients in Somalia. This study was a prospective cross-sectional study conducted on HC patients attended at Mogadishu Somali Turkish Training and Research Hospital in Mogadishu, Somalia, from November 2020 to April 2021. A total of 6239 patients were screened during the study period. The prevalence of HC was 2.1% (128/6239). Of them, 76 (59.4%) were males. The mean (SD) age of the participants was 56.5 (± 16.9) years (range: 24–98 years). 54.7% (70/128) met the criteria for a hypertensive emergency, while 45.3% (58/128) met the criteria of hypertensive urgency. Most patients (55.5%) took a single antihypertensive medicine, with calcium channel blockers being the most frequently used (57.8%). Headache and palpitation were the most often reported symptoms upon admission (39.1% and 25%). The most often prescribed antihypertensive drugs for the initial therapy included Intravenous furosemide (35.2%), Sublingual captopril (25.8%), intravenous nitroglycerin (23.4%), and intravenous labetalol (20%). Among the forms or patterns of end-organ damage of HE, we most frequently observed acute heart failure (45.7%), acute pulmonary edema (29.9%), and acute renal injury (25.7%). Infrequent medical checkups, poor compliance with medications, poor compliance with exercise, positive family history of hypertension, and being male gender were significant predictors of HC, AOR = 20.312; p < 0.000, AOR = 7.021; p < 0.008, AOR = 6.158; p < 0.017, AOR = 3.545; p < 0.032, and AOR = 2.144; p = 0.001, respectively. In Somalia, the hypertensive crisis is common in this clinic population. Infrequent medical checkups, poor compliance with medications and exercise, positive family history of hypertension, and being male gender were significant predictors of HC.

## Introduction

Hypertension is one of the most common global non-communicable diseases of public health concern. It is associated with different risk factors, and complications include heart failure, coronary heart disease, peripheral vascular disease, stroke, and chronic kidney disease^1^. Worldwide, the prevalence of hypertension is estimated to be as much as 1 billion individuals, and approximately 7.1 million deaths per year may be attributable to hypertension and its complications^2^. With an estimated 46 percent prevalence, it is one of the significant health issues in Africa^3^.

A hypertensive crisis is a life-threatening severe clinical situation in which an abrupt rise in arterial blood pressure (systolic blood pressure levels > 180 mmHg and/or diastolic blood pressure levels > 120 mmHg) can lead to acute damage of vital organs^4,5^. Hypertensive crises are classified as hypertensive emergencies in the presence of end-organ damage, including hypertensive encephalopathy, acute left ventricular failure combined with aortic dissection, different forms of arterial hypertension combined with subarachnoid bleeding or ischemic stroke, or hypertensive urgency in the absences of vital organ damage evident^5^. The hypertensive crises incidence/prevalence is rarely discussed in the medical literature. It has been estimated that around 1% of patients with hypertension develop a hypertensive crisis during their lifetime due to inadequate blood pressure (BP) control^3^. The prevalence of HC in Sub-Sahara African countries varied according to the population studied. It has been reported that around 2.5–13.2%^6,7^.

Patients' noncooperation, inappropriate therapy, endocrine illnesses, renal disease, pregnancy, and intoxication with drugs like methamphetamine and cocaine are the leading triggers of hypertensive crises^8^. In addition, early diagnosis, lifestyle modifications, and flowing guidelines of convenient antihypertensive drug therapy are essential to reduce the risk of hypertension-related morbidity and mortality^9^. Therefore, early diagnosis and proper management are needed to reduce the morbidity and mortality rate of cardiovascular disease and prevent the complications of hypertension.

As far as we know, there is no literature or study on the clinical–epidemiological profile and risk factors of patients with a hypertensive crisis in emergency services in Somalia. Therefore, the main objective of the present study was to determine the epidemiological profile, clinical characteristics, and risk factors of hypertensive crisis among patients admitted to the emergency unit of a tertiary hospital in Mogadishu, Somalia.

## Methods

### Study population, design, and setting

This study was a prospective cross-sectional study conducted on hypertensive patients who attended the Emergency clinic of Mogadishu Somali Turkish Training and Research Hospital; in Mogadishu, Somalia, from November 2020 to April 2021. It is the only teaching and referral hospital in Somalia with 225 inpatient beds, 21 adult ICU beds, 6 pediatric ICU beds, 19 neonatal ICUs, and more than 15 departments. The hospital delivers 24 h full service of emergency care service in its emergency department.

### Eligibility criteria

All adult patients who presented to our hospital's emergency care unit with a hypertension diagnosis during the study period were included. We excluded pregnant or lactating women, newly diagnosed hypertension, and participants with incomplete records. Patients who gave verbal consent for participation were also included in the study.

### Data collection and quality assurance

Before starting the study, a 5% sample pretest was done on randomly selected patients, and all nonsense questions were removed from the questionnaires accordingly. The questionnaire that has been tested and validated was employed. The principal investigator attentively assessed and ensured all data collection and recording processes and daily acquired data were documented and prepared for the next day's study.

Data were collected by an emergency medicine physician and supported by two trained resident doctors. The data was obtained in the form of questionnaires and medical records, including the patient's gender, age, marital status, educational level, duration of hypertension, any concurrent medical problems, laboratory investigations (e.g., complete blood count, liver and renal function tests, lipid profile, coagulation profile…etc.), lifestyle nature (e.g., physical activity, smoking habit, alcohol consumption, dietary habits, and etc.), any comorbidities, antihypertensive agents pattern, and any development of potential complications from the hypertensive.

The previous diagnosis of hypertension was identified through examination of the medical history or use of antihypertensive drugs.

A hypertensive crisis is defined as a systolic blood pressure level at > 180 mmHg and/or diastolic blood pressure level at > 120 mm in accordance with the guideline of the European Society of Hypertension^4^. All cases with one or more of the following types of acute end-organ damage (Hypertensive encephalopathy; congestive heart failure; acute pulmonary edema, acute myocardial infarction or unstable angina pectoris, and acute or progressive renal insufficiency) were classified as hypertensive emergencies. The conditions were diagnosed clinically and by diagnostic tests such as blood chemistry for serum creatinine and Urea, 12-lead electrocardiography, echocardiography, head computed tomography and magnetic resonance imaging, and ultrasound imaging as appropriate. In the absence of end-organ damage, all other hypertensive crises were considered by exclusion to be hypertensive urgencies. Hypertensive urgency was defined as high blood pressure that met the inclusion criteria but did not show signs of acute end-organ failure^10^.

An upper arm cuff automatic BP gadget was used by the emergency care nurse to assess blood pressure in the emergency room. Each individual's arm circumference was used to determine the appropriate cuff size. After 5 min of rest, at least two BP readings were taken at three-minute intervals with the patient in a supine or sitting posture.

### Data organization, presentation, and analysis

Data will be coded and entered into the Statistical Package for Social Science (SPSS) version 23 for Windows. Figures and tables were used to present the findings. The Chi-square test was used to compare proportions between patients with non-hypertensive crisis and hypertensive crisis. Kolmogorov–Smirnov test was used for testing the normality of continuous data. To compare continuous parametric and non-parametric data between the two groups (non hypertensive crisis and hypertensive crisis), the Student's *t* test and Mann–Whitney test were utilized. The hypertensive crisis was used as the dependent variable in regression analysis. If the univariate p was less than 0.20, independent variables (age, sex, marital status, education, presence of comorbidity, cigarette smoking, Khat chewing, physical activity, and family history of hypertension) were included in the model.

### Ethical considerations

This study was approved by the Clinical Research Ethics Committee of Mogadishu Somali Turkish Training and Research Hospital (Reference number: MSTH/10522). All methods were performed in accordance with the relevant Helsinki Declaration contents guidelines. Participants were informed about the purpose of the study, and written informed consent for participation was obtained from all participants.

## Results

### Prevalence, baseline characteristics, and comorbidities

A total of 6239 patients that presented to the medical emergency unit during the study period from November 2020 to April 2021 were screened. 128 patients did meet the criteria of hypertensive crises. Of them, 76 (59.4%) were males. The mean (SD) age of the participants was 56.5 (± 16.9) years (range: 24–98 years).

The prevalence of hypertensive crisis was 2.1% (128/6239) at the emergency department in Mogadishu Somali Turkish training and research hospital. 54.7% (70/128) met the criteria for a hypertensive emergency with elevated blood pressure 140/90 mmHg with associated target organ damage. In comparison, 45.3% (58/128) met the criteria of hypertensive urgency with BP more than 180/110 mmHg with no target organ damage.

The hypertensive emergency was found in 70 participants: 50 (71.4%) men and 20 (28.6%) women, while 58 examinees had hypertensive urgency: 26 (44.8%) men and 32 (55.2%) women. There was a significant statistical difference in the proportion of patients with hypertensive crisis to gender (p = 0.012).

Considering the participant age group, the majority of the examinees, 80 (62.5%) of them, belonged to the 40–69 age group, with 44 hypertensive emergency (62.9%) and 36 hypertensive urgency (62.1%). There was a significant statistical difference in the proportion of examinees with hypertensive crisis to gender (p = 0.012).

Regarding the duration of hypertension among patients with the hypertensive crisis, it was revealed that 54 patients (69.01%) had verified hypertension for 1–5 years. 32 (25%) patients had been treated for hypertension for a period of more than 10 years, 22 (17.2%) patients had been treated for 6–10 years, and 20 (15.6%) patients had hypertension for less than 1 year. There have been statistically significant differences in the proportion of examinees between these two groups (p = 0.001).

As shown in Table 1, the most common comorbidities at admission were diabetes (n = 58, 45.3%), dyslipidemia (n = 33, 25.8%), chronic kidney disease (n = 16, 12.5%), chronic obstructive pulmonary disease (n = 11, 8.6%), cardiovascular disease (n = 10, 7.8%), and chronic liver disease (n = 8, 6.3%).

**Table 1:** Baseline characteristics among patients with hypertensive crises.

Variables	Categories	Hypertensive crisis (n = 128, %)	Hypertensive emergency (n = 70, %)	Hypertensive urgency (n = 58, %)	p-value
Sex	Male	76 (59.4)	50 (71.4)	26 (44.8)	0.012
	Female	52 (40.6)	20 (28.6)	32 (55.2	
Age group (years)	18–39	22 (17.2)	12 (17.1)	10 (17.2)	0.011
	40–69	80 (62.5)	44 (62.9)	36 (62.1)	
	≥ 70	26 (20.3)	14 (20)	12 (20.7)	
Duration of hypertension by years	< 1	20 (15.6)	16 (22.9)	4 (6.9)	0.001
	1–5	54 (42.2)	32 (45.7)	22 (37.9)	
	6–10	22 (17.2)	14 (20)	8 (13.8)	
	> 10	32 (25)	8 (11.4)	24 (41.4)	
Comorbidities	Diabetes	58 (45.3)	30 (42.9)	28 (48.3)	0.540
	Dyslipidemia	33 (25.8)	21 (30)	12 (20.7)	0.002
	CKD	16 (12.5)	11 (15.7)	5 (8.6)	0.119
	COPD	11 (8.6)	8 (11.4)	3 (5.2)	0.381
	CVD	10 (7.8)	6 (8.6)	4 (6.9)	0.428
	CLD	8 (6.3)	8 (11.4)	0 (0)	0.022
	Thyroid dysfunction	6 (4.7)	4 (5.7)	2 (3.4)	0.546
	Malignancy	3 (2.3)	2 (2.9)	1 (1.7)	0.117

*CKD* chronic kidney disease, *COPD* chronic obstructive pulmonary disease, *CVD* cardiovascular disease, *CLD* chronic liver disease.

### Pattern and distribution of antihypertensive medications

**Figure 1:**
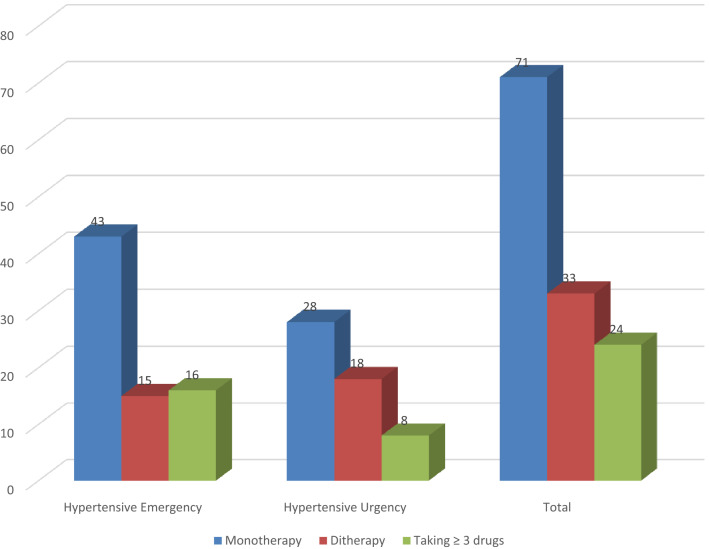
Pattern of antihypertensive medications.

**Figure 2:**
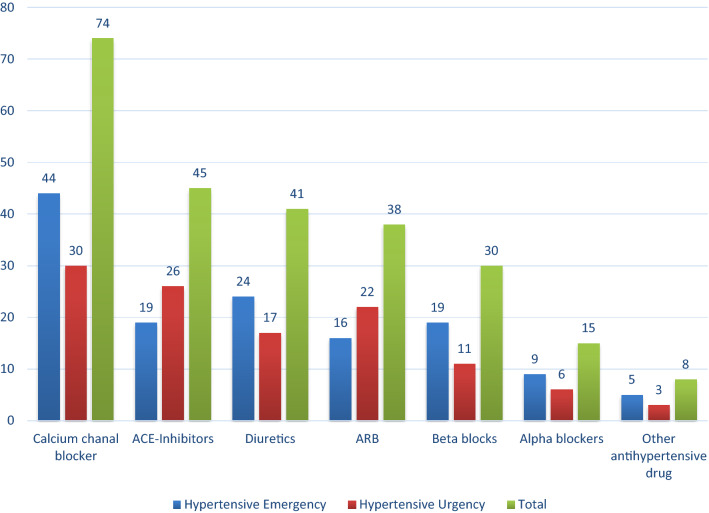
Distribution of antihypertensive medications.

All patients were taking at least one antihypertensive medication, with 71 (55.5%) examiners taking a single drug, 33 (25.8%) examiners taking a dual-drug combination, and 24 (18.8%) examiners taking three or more combined medications (Fig. 1). Antihypertensive treatment includes calcium channel blockers for 71 patients (55.5%), angiotensin-converting enzyme inhibitors for 45 patients (35.2%), diuretics for 41 patients (32%), angiotensin receptor blockers for 38 patients (29.7%), beta-blockers for 30 patients (23.4%), and alpha-blockers for 15 patients (11.7%). The distribution of drug types was different across the two groups, with calcium channel blockers, diuretics, and beta blockers being the most common medications for patients with a hypertensive emergency (Fig. 2).

### Risk factors

Risk factors for hypertensive urgency and emergency are summarized in Table 2. About twenty-six (20.3%) of enrolled patients were current smokers. In comparison, up to 20 (171%) patients reported a history of a smoker. More one two-thirds (n = 46, 35.9%) of the patients had either current or past history of khat chewing. 46 (35.9%) participants had a family history of hypertension. Regarding physical activity, only 40 (31.2%) participants had good compliance with regular exercise. Moreover, only 41 (32%) participants were on regular/frequent medical check-ups. Up to 42 (32.8%) participants performed diet control, while 43 (33.6%) participants were reasonably compliant with medications. According to the results presented in Table 2, there is a statistically significant difference in the regular medications and regular medical check-ups between the hypertensive emergency and hypertensive urgency (p < 0.05).

**Table 2:** Risk factors for patients with hypertensive crises.

Variables	Categories	HC, n = 128 (%)	HE, n = 70 (%)	HU, n = 58 (%)	p-value
Smoking	Never smoker	82 (64.1)	44 (62.9)	38 (65.5)	0.260
	Current smoker	26 (20.3)	14 (20)	6 (10.3)	
	Past smoker	20 (15.6)	12 (17.1)	14 (24.1)	
Khat chewing	Never chew	82 (64.1)	44 (62.9)	38 (65.5)	0.399
	Current chewer	25 (19.5)	18 (25.7)	10 (17.2)	
	Past chewer	21 (16.4)	8 (11.4)	10 (17.2)	
Family history of HTN	Yes	46 (35.9)	28 (40)	18 (31)	0.293
	No	82 (64.1)	42 (60)	40 (69)	
Compliance with exercise	Good	40 (31.2)	18 (25.7)	22 (37.9)	0.138
	Poor	88 (68.8)	52 (74.3)	36 (62.1)	
Compliance with medication	Good	43 (33.6)	9 (12.9)	34 (58.6)	0.000
	Poor	85 (66.4)	61 (87.1)	24 (41.4)	
Performing diet control	Yes	42 (32.8)	22 (31.4)	20 (34.5)	0.714
	No	86 (67.2)	48 (68.6)	38 (65.5)	
Medical checkup	Frequent	41 (32)	12 (17.1)	29 (50)	0.001
	Infrequent	87 (68)	58 (82.9)	29 (50)	

### Clinical presentations

The most common presenting symptoms at admission were headache (n = 50, 39.1%), palpitation (n = 32, 25%), chest pain (n = 32, 25%), dyspnea (n = 31, 24.2%), vomiting (n = 28, 21.9%), altered level of conscious (n = 24, 18.8%), epigastric pain (n = 24, 18.8%), and focal neurologic deficits (n = 22, 17.2%). The four most significant symptoms among patients with hypertensive emergencies were palpitation (n = 23, 32.9%; p = 0.031), Chest pain (n = 20, 28.6%; p = 0.024), altered level of consciousness (n = 19, 27.1%; p = 0.008), and focal neurologic deficits (n = 18, 25.7%; p = 0.005), while the three most common significant initial features among patients with hypertensive urgency were vomiting (n = 16, 27.6%; p = 0.032), epigastric pain (n = 16, 27.6%; p < 0.020), and syncope (n = 6, 10.3%; p = 0.006) (Table 3).

**Table 3:** Initial clinical presentation of hypertensive crises patients.

Clinical presentations	HC, n = 128 (%)	HE, n = 70 (%)	HU, n = 58 (%)	p-value
Headache	50 (39.1)	27 (38.6)	23 (39.7)	0.900
Palpitation	32 (25)	23 (32.9)	8 (13.8)	0.031
Chest pain	32 (25)	20 (28.6)	12 (20.7)	0.024
Dyspnea	31 (24.2)	18 (25.7)	13 (22.4)	0.664
Vomiting	28 (21.9)	12 (17.1)	16 (27.6)	0.032
ALOC	24 (18.8)	19 (27.1)	5 (8.6)	0.008
Epigastric pain	24 (18.8)	8 (11.4)	16 (27.6)	0.020
Focal neurologic deficities	22 (17.2)	18(25.7)	4 (6.9)	0.005
Oliguric	7 (5.5)	5 (7.1)	2 (3.4)	0.360
Dizziness	7 (5.5)	4 (5.7)	3 (5.2)	0.893
Syncope	6 (4.7)	0 (0)	6 (10.3)	0.006
Other clinical features	12 (9.4)	7 (10)	5 (8.6)	0.267
Asymptomatic	5 (3.9)	2 (2.9)	3 (5.2)	0.857

*HC* hypertensive crisis, *HE* hypertensive emergency, *HU* hypertensive urgency.

### Initial management and complications

As shown in Table 4, intervenous furosemide was the most commonly used antihypertensive medication for the initial management of our patients with hypertensive crisis (35.2%), followed by Sublingual captopril (25.8%), intervenous nitroglycerin (23.4%), intervenous labetalol (20%), intervenous esmolol (10%), and sublingual nitroglycerin (7%). There was no significant statistical difference in the proportion of patients with hypertensive crisis to initial medications.

**Table 4:** Initial therapy of choice among patients with hypertensive crisis.

Medications	HC, n = 128 (%)	HE, n = 70 (%)	HU, n = 58 (%)	p-value
Intervenous furosemide	45 (35.2)	19 (27.1)	26 (44.8)	0.496
Sublingual captopril	33 (25.8)	19 (27.1)	14 (24.1)	0.542
Intervenous nitroglycerin	30 (23.4)	18 (25.7)	12 (20.9)	0.865
Intervenous labetalol	20 (15.6)	14 (20)	6 (10.3)	0.385
Intervenous esmolol	13 (10.2)	7 (10)	6 (10.3)	0.612
Sublingual nitroglycerin	9 (7)	5 (7.1)	4 (6.9)	0.231

*HC* hypertensive crisis, *HE* hypertensive emergency, *HU* hypertensive urgency.

**Figure 3:**
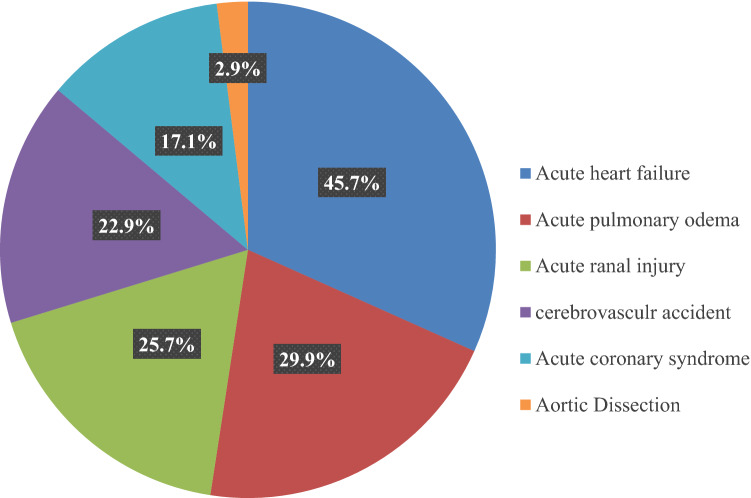
Pattern of target organ damages of patients with hypertensive emergencies.

According to the Pattern of target organ damages of patients with hypertensive emergencies, 32 (45.7%) participants had evidence of acute cardiac failure, 23 (29.9%) participants had acute pulmonary edema, 18 (25.7%) participants had an acute renal injury, 16 (22.9%) participants had evidence of a cerebrovascular accident, 12 (17.1%) participants had evidence of acute coronary syndrome, and only two (2.9%) participants had evidence of aortic dissection (Fig. 3).

Multiple logistic regression model was used. After adjustment for the other variables, gender, family history of hypertension, performing any exercise, regular medication use, and regular medical check-up were significantly predicting the hypertensive crisis among hypertensive patients. We found that male patients were two times higher of having hypertensive crisis compared with females (AOR = 2.144; p = 0.001), having a family history of hypertension were 3.5 times higher of being hypertensive crisis compared with no family history of hypertension (AOR = 3.545; p < 0.032), for those who had poor compliance with exercise were 7.021 times higher of being hypertensive crisis comparing to those with poor compliance to exercise AOR = 6.158; p < 0.017. Moreover, we conducted those patients with poor compliance to the medications were 7.021 times higher being hypertensive crisis compared to those with poor adherence to exercise (AOR = 7.021; p < 0.008), and those with infrequent medical check-ups were 20.312 times higher being hypertensive crisis compared those with regular/frequent medical check-up (AOR = 20.312; p < 0.000) (Table 5).

**Table 5:** Predictors of hypertensive crisis.

Variables	Hypertensive crisis, n = 128 (%)	Odd ratio	95% CI		p value
**Gender**					
Male	76 (59.4%)	2.144	1.460	4.645	0.001
Female	52 (40.6%)	1			
**Family hx of HTN**					
Yes	46 (35.9%)	3.545	1.345	6.905	0.032
No	2 (64.1%)	1			
**Compliance with exercise**					
Poor	40 (31.2%)	1			
Good	88 (68.8%)	6.158	1.738	27.530	0.017
**Compliance with medication**					
Poor	43 (33.6%)	1			
Good	85 (66.4%)	7.021	2.418	17.620	0.008
**Medical checkup**					
Frequent	41 (32%)	1			
Infrequent	87 (68%)	20.312	5.521	75.130	0.000

## Discussion

A hypertensive crisis is a life-threatening severe clinical condition in which an abrupt rise in arterial blood pressure (systolic blood pressure levels > 180 mmHg and/or diastolic blood pressure levels > 120 mmHg) that can lead to acute damage of vital organs^4,5^. Particularly in low-income nations, the early diagnosis and management of hypertensive crisis provide a specific difficulty^10^.

The prevalence of hypertensive crisis was 2.1% (128/6239), with 54.7% (70/128) met the criteria for a hypertensive emergency. In comparison, 45.3% (58/128) met the criteria of hypertensive urgency. The findings of the prevalence of hypertensive crisis in our study are slightly lower than the prevalence reported from Tanzania (2.5%)^7^. Additionally, the prevalence of hypertensive crisis reported from Uganda (5.1) was much higher than our findings^11^.

The prevalence reported from earlier studies in Africa by Ellenge et al. and Garcia et al. showed a prevalence of 4–4.3%, two times higher than the current study's findings^12,13^. This can be because the study environments varied.

In the present study, despite the risk for death and the seriousness of the hypertensive emergency, this condition was the most common type of hypertensive crisis when compared to hypertensive urgency, 54.7% (n = 70) and 45.3% (n = 58), respectively. These findings contrast with a multicenter study conducted in Italy, where the prevalence of hypertensive urgency and emergency was 74.7% and 25.3%, respectively^14^.

Using the operational classification of hypertensive crisis in urgencies and emergencies, data obtained from Ethiopia discovered that hypertension urgencies (65.9%) were more common than hypertensive emergencies among 252 patients with hypertensive crisis^15^.

In this regard, comparable findings were reported from Tanzania, Congo, Uganda, and East Sudan, with a high proportion of hypertensive emergencies at 68%, 76%, 67.5%, and 61.7%, respectively^7,16–18^.

There are numerous reasons why patients with hypertensive emergencies are more common in this setting, including lack of knowledge to manage hypertension, lack of awareness about control of hypertension, poor adherence to antihypertensive medications, and the difficulty of the poor to obtain healthcare may also be at fault.

Previous studies highlighted an inconsistency in gender distribution among patients admitted to the EDs for hypertensive crises, with a higher proportion of women than men in contrast to our research^19,20^.

On the other hand, the current study revealed that men were significantly associated with HC; this may be attributed to the differences in hemodynamic status between women and men, as women, until menopause, have lower peripheral vascular resistance. Hence, they have lower blood pressure levels than men of the same age.

In addition, the present study showed a significant statistical difference in the proportion of examinees with hypertensive crisis to gender (p = 0.012). Similarly, in the current study, Rashed and his collagenous reported that there were more men than women in their work (100:54) and that an abundant number of the patients belonged to the 45–65 years of age group^21^.

Most patients in our study self-reported risk factors for the hypertensive crisis included; Cigarette smoking, poor compliance with exercise, infrequent medical checkups, poor diet control, and poor compliance with medication. Moreover, there is a statistically significant difference between the hypertensive emergency and hypertensive urgency in the regular medications and regular medical checkups (p < 0.05). Correspondingly to our study, previous studies have mentioned that the most important precipitating factors of a hypertensive crisis in known hypertensive patients are obesity, history of hypertension, low socioeconomic status, poor health literacy, cigarette smoking, lack of physical exercise, sedentary work, poor adherence and compliance to antihypertensive medication procedures^22–25^.

The signs and symptoms presented on admission to the hospital vary according to the clinical presentation of a hypertensive crisis. In our study, the headache was the most common initial clinical feature, followed by palpitation, chest pain, dyspnea, vomiting, and altered level of consciousness. These results are consistent with most earlier studies^19,20,26^. These symptoms could indicate a wide range of possible diagnoses and highlight the need for a thorough evaluation before disposition.

All patients received at least one antihypertensive treatment, with 71 (55.5%) examiners using a single medication, 33 (25.8%) patients taking dual medicines, and 24 (18.8%) patients taking three or more mixed medications. According to a similar study from Ethiopia, the most prevalent category of the antihypertensive drug was monotherapy users (n = 85, 51.2%), followed by dual drug users (n = 51, 30.7%), while only thirty (18%) patients used three or more combination drugs^15^.

The antihypertensive treatment prescribed includes calcium channel blockers (n = 71, 55.5%), angiotensin-converting enzyme inhibitors (n = 45, 35.2%), diuretics (n = 41, 32%), angiotensin receptor blockers (n = 38, 29.7%), and beta-blockers (n = 30, 23.4%). This finding was in agreement with the study reports from Ethiopia^15^.

In our institution, intravenous furosemide, sublingual captopril, intervenous nitroglycerine, and intervenous labetalol administrations were preferred for managing hypertensive crises in patients requiring prompt blood pressure control as a treatment approach. In contrast to this finding, a study from northwest Ethiopia reported oral captopril, intervenous hydralazine, oral nifedipine, oral enalapril, and oral hydrochlorothiazide as the most frequently administered medication by hypertensive crisis patients^15^. A shortage of available intravenous medicines and physician reluctance to rapidly decrease blood pressure may be responsible for this difference.

Based on the data of our study on the initial target organ damage of hypertensive emergency in the emergency unit, acute cardiac failure was the most common, followed by acute pulmonary edema, acute kidney injury, and cerebrovascular accident, in disagreement with various studies^11–13^. In comparison with a study conducted in Sudan by Abdallah et al., it was shown that hemorrhagic stroke was the most frequent target organ damage among patients with a hypertensive emergency, followed by ischemic stroke, left ventricular failure, acute coronary syndrome, and renal failure^18^. But acute coronary syndrome was rare in the study (9.4%), and a similar finding was reported in other African countries^7,12^.

## Strengths and limitations of the study

Despite the importance of the topic, to the best of our knowledge and a literature search, this is the first study reporting the clinic-epidemiological profile and risk factors of hypertensive crisis in an ED population in Somalia.

Our findings are original, as no previous epidemiological study has estimated the rate of hypertensive crises on hospital admissions using standardized data collection methods in Somalia. The main objective of our study was to determine the prevalence, patterns, and factors associated with hypertensive crises in the emergency department of a single tertiary hospital in Somalia.

The study's primary weakness was that the results of this study cannot be extended to the entire nation of Somalia and beyond because it was carried out at a single center in a specific section of the country. Second, the study was further constrained by the small sample size, which might have prevented the detection of meaningful distinctions between hypertensive urgency and emergency.

## Conclusion

The present study shows that the prevalence of hypertensive crises accounted for 2.1% of medical admissions in the emergency department. Moreover, the majority of cases were hypertensive emergencies (54.7%). Infrequent medical checkups, poor compliance with medications and exercise, positive family history of hypertension, and being male gender were significant predictors of hypertensive crises.

Even though most patients did not receive guideline-recommended treatment, to improve compliance with treatment, health professionals should educate the patients. Further research is required to determine the etiology, pathophysiology, and most appropriate strategies for preventing hypertensive crisis.

## Data Availability

The datasets used and/or analyzed during the current study are available from the corresponding author (Mohamed Farah Yusuf Mohamud: m.qadar59@gmail.com) upon reasonable request.
